# Bacterial ribosomal RNA detection in cerebrospinal fluid using a viromics approach

**DOI:** 10.1186/s12987-022-00400-5

**Published:** 2022-12-22

**Authors:** Cormac M. Kinsella, Arthur W. D. Edridge, Ingeborg E. van Zeggeren, Martin Deijs, Diederik van de Beek, Matthijs C. Brouwer, Lia van der Hoek

**Affiliations:** 1 Amsterdam UMC, Laboratory of Experimental Virology, Department of Medical Microbiology and Infection Prevention, University of Amsterdam, Meibergdreef 9, 1105 AZ Amsterdam, The Netherlands; 2 Amsterdam Institute for Infection and Immunity, Postbus 22660, 1100 DD Amsterdam, The Netherlands; 3 Amsterdam UMC, Department of Neurology, University of Amsterdam, Meibergdreef 9, 1105 AZ Amsterdam, The Netherlands; 4 Amsterdam Neuroscience, Neuroinfection and Inflammation, Amsterdam, The Netherlands

**Keywords:** Bacterial meningitis, Pathogen detection, Cerebrospinal fluid, Viromics, Metagenomics

## Abstract

**Background:**

In patients with central nervous system (CNS) infections identification of the causative pathogen is important for treatment. Metagenomic next-generation sequencing techniques are increasingly being applied to identify causes of CNS infections, as they can detect any pathogen nucleic acid sequences present. Viromic techniques that enrich samples for virus particles prior to sequencing may simultaneously enrich ribosomes from bacterial pathogens, which are similar in size to small viruses.

**Methods:**

We studied the performance of a viromic library preparation technique (VIDISCA) combined with low-depth IonTorrent sequencing (median ~ 25,000 reads per sample) for detection of ribosomal RNA from common pathogens, analyzing 89 cerebrospinal fluid samples from patients with culture proven bacterial meningitis.

**Results:**

Sensitivity and specificity to *Streptococcus pneumoniae* (n = 24) before and after optimizing threshold parameters were 79% and 52%, then 88% and 90%. Corresponding values for *Neisseria meningitidis* (n = 22) were 73% and 93%, then 67% and 100%, *Listeria monocytogenes* (n = 24) 21% and 100%, then 27% and 100%, and *Haemophilus influenzae* (n = 18) 56% and 100%, then 71% and 100%. A higher total sequencing depth, no antibiotic treatment prior to lumbar puncture, increased disease severity, and higher c-reactive protein levels were associated with pathogen detection.

**Conclusion:**

We provide proof of principle that a viromic approach can be used to correctly identify bacterial ribosomal RNA in patients with bacterial meningitis. Further work should focus on increasing assay sensitivity, especially for problematic species (e.g. *L. monocytogenes*), as well as profiling additional pathogens. The technique is most suited to research settings and examination of idiopathic cases, rather than an acute clinical setting.

**Supplementary Information:**

The online version contains supplementary material available at 10.1186/s12987-022-00400-5.

## Key points

We demonstrate that an unmodified viromic next-generation sequencing methodology effectively detects bacterial pathogen ribosomal RNA in cerebrospinal fluid samples. Sensitivity and specificity displayed pathogen dependent variation. Our finding in principle offers avenues for simultaneous detection of neuropathogenic viruses and bacteria.

**Supplementary Information:**

The online version contains supplementary material available at 10.1186/s12987-022-00400-5.

## Introduction

In patients with central nervous system (CNS) infections, rapid identification of the causative pathogen is essential to inform treatment and improve prognosis [1, 2]. The differential diagnoses in these patients may include auto-immune disease, non-infectious neurological disease, or non-neurological infection [3, 4]. Clinical characteristics fail to adequately differentiate between potential causes, therefore microbiological testing on cerebrospinal fluid (CSF) is the cornerstone of diagnosing CNS infections [5]. Currently available diagnostics include antigen/antibody detection assays, direct microscopy, culture techniques, and quantitative polymerase chain reaction (qPCR). Despite the availability of these tests, in a substantial proportion of patients with a high suspicion of CNS infection, no infectious organism can be identified. Because conventional assays often target specific, common pathogens, uncommon or unknown pathogens may be missed [6, 7]. Metagenomic next-generation sequencing (mNGS) is an emerging technique to diagnose CNS infection without targeting specific pathogens [8, 9], and is theoretically capable of identifying any pathogen RNA or DNA in samples. As sensitivity and specificity of mNGS assays have yet to match conventional testing, further development is warranted [10, 11].

For detection of viral pathogens, specialized ‘viromic’ mNGS methods have been developed in recent years. Viromic techniques apply mNGS to clinical samples enriched for virus-like particles, minimizing sequencing of host and background nucleic acids in order to maximize sensitivity to viruses. Virus discovery cDNA-amplified fragment length polymorphism (VIDISCA) is one such viromic assay that enables broad detection of known viruses, and has also been applied in the discovery of many novel eukaryotic viruses [12–16]. Viromic assays begin with centrifugation to remove cellular material while retaining virions in supernatant. VIDISCA then treats supernatant with DNase enzymes to remove residual genomic DNA (gDNA), which is unprotected—unlike most viral DNA. Neither step will remove residual mRNA or ribosomes, the latter of which are equivalent in size to small viruses and often highly abundant, depending on the sample type. Consequently, a high proportion of sequence data from clinical specimens can consist of ribosomal RNA (rRNA), which hinders virus detection via competition. To avoid this, VIDISCA incorporates a reverse transcription step using custom hexamer primers that mostly cannot anneal to mammalian rRNA [17], reducing human rRNA sequence reads by over 90% [18]. We previously observed these hexamers still bind to rRNA of some eukaryotic parasites [15], increasing the diagnostic capacity of VIDISCA. So far, the detection of prokaryotic pathogens has not been described. Here, we evaluated the performance of VIDISCA in detection of bacterial rRNA in CSF samples from patients with culture proven bacterial meningitis.

## Methods

### Sample description

Patients participated in the MeninGene study, a nationwide prospective cohort study of community-acquired bacterial meningitis in the Netherlands, methods of which have been described elsewhere [19, 20]. Briefly, patients with a positive CSF culture were identified by the Netherlands Reference Laboratory for Bacterial Meningitis (NRLBM), which receives the cultured pathogen from 85% of bacterial meningitis patients in the Netherlands. The NRLBM notified the researchers, who contacted the treating physician, who subsequently informed patients or their legal representative about the study. Patients could also be included by their treating physician without notification by the NRLBM. All patients or representatives gave written informed consent, and the study was approved by the Medical Ethics Committee of the Amsterdam UMC (METC2013_043). Clinical data were collected using an online case record form and patient outcome was recorded using the Glasgow Outcome Scale [21]. Leftover CSF was stored at treatment centers at − 80 °C and transferred to the Amsterdam UMC biobank facility. For this study, 89 CSF samples with sufficient residual material were selected. Researchers performing library preparation, sequencing and metagenomic analysis were blinded to patient clinical information and the diagnosed pathogen. Subsequently, data were unblinded for optimization of threshold parameters. For controls, previously generated [22] sequencing data from 74 patient CSF samples tested negative in culture for bacteria were included in analysis, approved by a separate decision of the Medical Ethics Committee of the Amsterdam UMC (METC2014_290). These samples were from patients undergoing lumbar puncture for suspected CNS infection, and either viral CNS infection was diagnosed or CNS infection was eventually ruled out.

### Library preparation and sequencing

VIDISCA library preparation was performed on CSF as previously described [14]. Briefly, 110 µl of CSF was centrifuged for 10 min at 5000 g, and supernatant was treated with TURBO DNase (Thermo Fisher Scientific) for 30 min at 37 °C. Nucleic acids were extracted using the Boom method [23], followed by reverse transcription primed with non-ribosomal hexamers [17] and second strand synthesis using Klenow fragment (3′ → 5′ Exo-, NEB). After clean-up by phenol/chloroform extraction and ethanol precipitation, dsDNA was digested with Mse1 (NEB) and ligated to sample specific adapters. Size selection with AMPure XP beads (Agencourt) was done to remove small DNA fragments. After a 28-cycle PCR, further size selection was done to retain fragments 200–600 bp long. Library concentrations were analysed using the Qubit dsDNA HS Assay Kit (Thermo Fisher Scientific), and pooled at equimolar concentration. Pool concentration and fragment length distribution were analysed using the Qubit and Bioanalyzer (High Sensitivity Kit, Agilent Genomics) instruments respectively. Sequencing was carried out on the Ion S5 System with the Ion 510 Chip Kit (Thermo Fisher Scientific).

### Metagenomic analyses

For bacterial rRNA identification, reads were mapped to the SILVA 138.1 SSU and LSU NR99 rRNA databases [24] using BWA MEM v0.7.17-r1188 [25]. Outputs were processed using the PathoID module of PathoScope v2.0.7 [26], and hits to phylum Chordata were removed. Remaining reads were realigned to the GenBank nt database using BLASTn [27], and hits to the five bacterial pathogens included in this study were counted. Blinded diagnostic predictive ability was explored by selecting the pathogen with the highest read count per sample as the predicted species, and those with equal counts as indeterminate. Unblinded detection performance per pathogen was then measured via sensitivity and specificity calculations. This was repeated varying several threshold parameters to understand their impact and optimize detection performance; parameters were minimum pathogen specific read count (≥ 1 read versus ≥ 10 reads), pathogen read identity to reference (≥ 97% versus 100%), and sample sequencing depth (all samples versus samples ≥ 10,000 reads). The level of human background per sample was estimated by mapping reads to a human rRNA database, subsetted from the aforementioned SILVA database. Hits were realigned to the same database using BLASTn, with reads retained and counted if they matched with 100% nucleotide identity for at least 100 bp.

Sources of bacterial reads (rRNA versus non-rRNA) were assessed by mapping them to genome assemblies of *Streptococcus pneumoniae* (GCF_002076835.1), *Neisseria meningitidis* (GCF_008330805.1)*, Listeria monocytogenes* (GCF_000196035.1)*, Haemophilus influenzae* (GCF_004802225.1), and *Klebsiella pneumoniae* (GCF_000240185.1). This was done first using original assemblies, and then versions with rRNA genes masked by RepeatMasker v4.1.1 [28]. Reads were curated by realignment to the respective original reference using BLASTn (requiring 100% identity for ≥ 100 bp). The rRNA count was calculated by subtracting the masked assembly read count from the original assembly read count. To determine if non-rRNA was predominately from mRNA or intact gDNA, the proportion mapping to reference coding sequences from each pathogen was calculated, with quality filtration as above. The proportion of coding sequence reads was compared with the gene density of each genome (coding sequence length/total genome length), since this represents the expected proportion of coding sequence reads derived from randomly sequenced pure gDNA.

Detection of viruses was done using a simplified version of a previously published workflow [29]. Reads were aligned to a database of viral proteins, with hits realigned to the GenBank nt database using BLASTn. Those aligning to non-viral sequences were removed as false positives, while those aligning either to viruses or to no reference were retained for manual examination. Reads from viruses of interest were aligned to respective reference genomes (Enterovirus D68: AY426531.1, HIV-1: AF286365.1) in Geneious v4.8.5 (https://www.geneious.com) to visualize genomic position and coverage. HIV-1 reads were subtyped using the Los Alamos National Laboratory HIV BLAST tool (www.hiv.lanl.gov).

### Statistical testing

We explored whether clinical characteristics were associated with detection of bacteria using Fisher’s exact tests for dichotomous data or Mann–Whitney U tests for continuous data. Sequencing depth was correlated to clinical characteristics using Spearman’s rank correlation. A p-value of < 0.05 was considered statistically significant.

## Results

### Patient characteristics

Between 2006 and 2022, 2705 patients with bacterial meningitis were included in the MeninGene study. We selected 89 patient samples from this cohort for VIDISCA analysis (Additional file 2: Table S1). Culture based pathogen diagnoses were *S. pneumoniae* (n = 24), *N. meningitidis* (n = 22), *L. monocytogenes* (n = 24), *H. influenzae* (n = 18), and *K. pneumoniae* (n = 1). Thirty-nine patients were female (44%) and the median age was 58 years (interquartile range (IQR) 34–67). Nine patients (10%) were being treated with antibiotics at the moment of presentation. The most frequent symptoms on presentation were fever in 72 patients from 81 case record reports (89%) and headache in 65 patients from 81 reports (80%). An altered mental status, defined as a Glasgow Coma Scale (GCS) score < 14, was seen in 47 patients (53%). Ten patients (11%) were in a comatose state (GCS < 8) upon presentation. Median number of CSF leukocytes was 2560/mm^3^ (IQR 768–5680) with a median CSF total protein concentration of 3.1 g/L (IQR 1.8–5.5). Seventy-three patients (82%) had a favorable outcome, defined as a score of five on the Glasgow Outcome Scale at discharge, and six died (7%). The negative controls consisted of 38 patients with viral CNS infection and 36 patients with initial suspicion of CNS infection, eventually ruled out.

### VIDISCA performance in bacterial diagnostics

All 89 CSF samples successfully yielded VIDISCA sequencing reads, with a median of 24,706 (IQR 10,151–40,320). From this we took the lower quartile value (rounded to 10,000 reads) as a threshold sequencing depth, to assess whether bacterial detection was affected by total sequencing depth. Under permissive parameters (any read depth, ≥ 1 pathogen specific read, and ≥ 97% read identity) bacterial pathogen reads were identified in 65 of 89 samples (73%), though only 43 contained reads from a single species. Under strict parameters (sample total read depth ≥ 10,000, ≥ 10 pathogen specific reads, and 100% read identity) 37 of 67 samples (55%) met the pathogen read threshold. Selecting the pathogen with the highest read count per sample, for permissive parameters the culture diagnosed pathogen was correctly predicted for 45 of 65 (69%) samples with bacterial reads, or 51% of all 89 samples. In 19 samples an incorrect pathogen was predicted (21% of 89), all of which were predicted as *S. pneumoniae*, showing this pathogen carries a particular risk of false positive identification. The final sample with bacterial reads was indeterminate. Under strict parameters the culture diagnosed pathogen was correctly predicted in 34 of 37 (92%) samples, or 38% of all 89, with incorrect prediction for 3 of 89 (3%) samples.

**Fig. 1:**
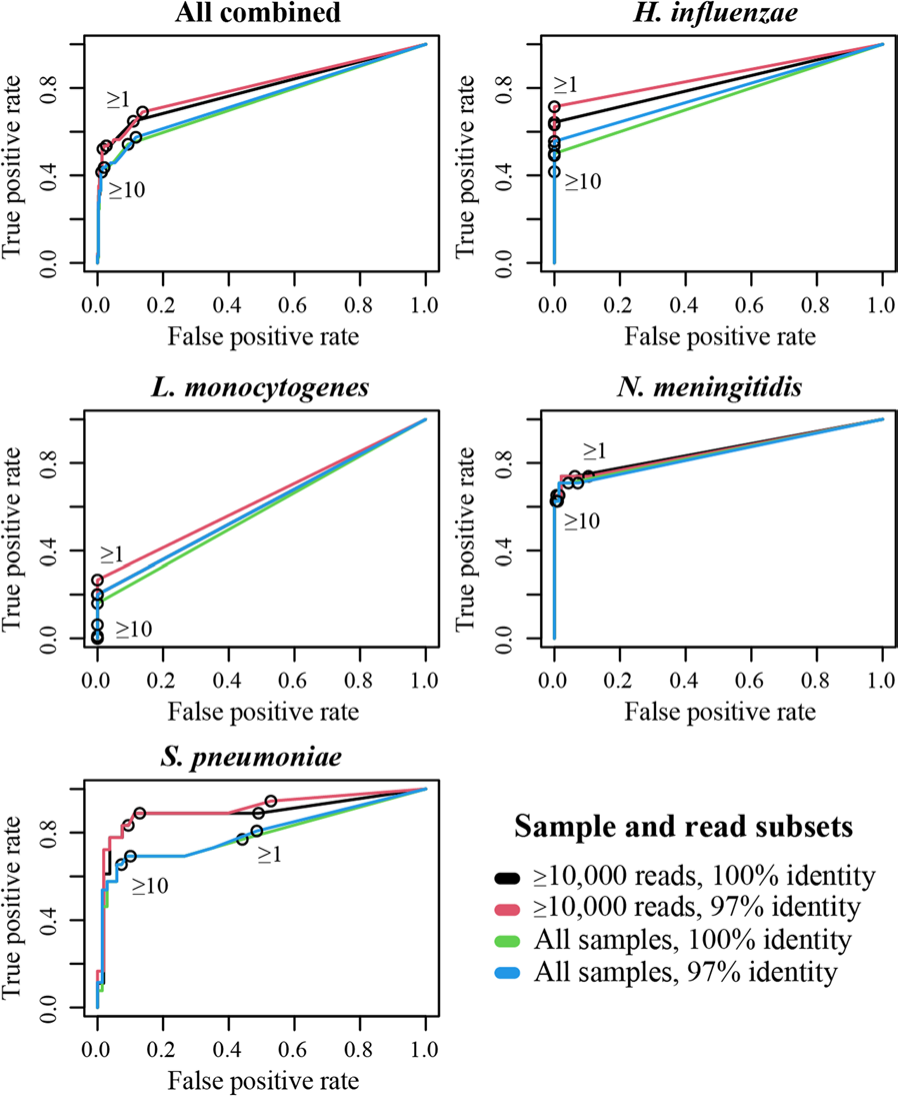
Receiver operating characteristic curves showing VIDISCA diagnostic performance with various parameters set. All 89 samples are analysed together (top left), and then separately by pathogen. *K. pneumoniae* is not shown, because n = 1. Subset key refers to total sequencing depth required for sample inclusion, and minimum read alignment identity to a bacterial reference sequence (for ≥ 100 nt). Numbers within charts refer to minimum number of pathogen reads identified for a sample to be called positive

We next explored the impact of threshold parameters on overall and per pathogen diagnostic performance. Alignment identity cut-off, sequencing depth, and pathogen specific read count impacted test diagnostic accuracy, with overall sensitivity to any pathogen ranging from 40% (30–51% CI) to 69% (56–79% CI), and specificity from 87% (82–90% CI) to 99% (97–100% CI; Fig. 1, Additional file 2: Table S2). For individual pathogens, there was high sensitivity to *S. pneumoniae*, *N. meningitidis*, and *H. influenzae* across parameters, and poor sensitivity to *L. monocytogenes* (Fig. 1, Table 1). The single *K. pneumoniae* positive sample was successfully detected across all parameter thresholds. Specificity was high across pathogens, with the exception of certain parameter thresholds for *S. pneumoniae*. Diagnostic performance was higher when only samples with ≥ 10,000 reads were considered, driven by increased sensitivity at low specificity cost, though the impact varied from minimal (e.g. *N. meningitidis*) to large (e.g. *S. pneumoniae*) (Fig. 1). For minimum pathogen read count, requiring ≥ 10 reads reduced false positive rates for *S. pneumoniae* from ~ 50% to ~ 10% when compared to ≥ 1 read (Fig. 1). The impact was lower for *L. monocytogenes, H. influenzae,* and *N. meningitidis,* which already had low false positivity rates at the ≥ 1 read threshold (≤ 11%). A ≥ 1 read threshold increased sensitivity for all pathogens compared with ≥ 10 reads, particularly for *L. monocytogenes* and *H. influenzae*. Alignment identity requirement had minimal overall impact, though in some cases the ≥ 97% cut-off increased sensitivity compared to the 100% cut-off at low to no specificity cost (Fig. 1), leading us to select this as the universal cut-off.

**Table 1:** Diagnostic performance per pathogen

Pathogen	Pathogen specific reads^a^	Total sample read depth (N)^b^	Sensitivity	Specificity
*H. influenzae*	≥ 1 read	All (18)	56%	100%
		≥ 10,000 reads (14)	71%	100%
*K. pneumoniae*	≥ 10 reads	All (1)	100%	100%
		≥ 10,000 reads (1)	100%	100%
*N. meningitidis*	≥ 10 reads	All (23)	64%	100%
		≥ 10,000 reads (22)	67%	100%
*S. pneumoniae*	≥ 10 reads	All (24)	67%	92%
		≥ 10,000 reads (16)	88%	90%
*L. monocytogenes*	≥ 1 read	All (24)	21%	100%
		≥ 10,000 reads (15)	27%	100%

^a^For each pathogen, the minimum pathogen read count with the best diagnostic performance is reported. ^b^Performance was compared between all samples and the subset with total read count ≥ 10,000. The 97% read identity dataset was used. See Additional file 2: Table S2 for performance at alternative threshold parameters

**Fig. 2:**
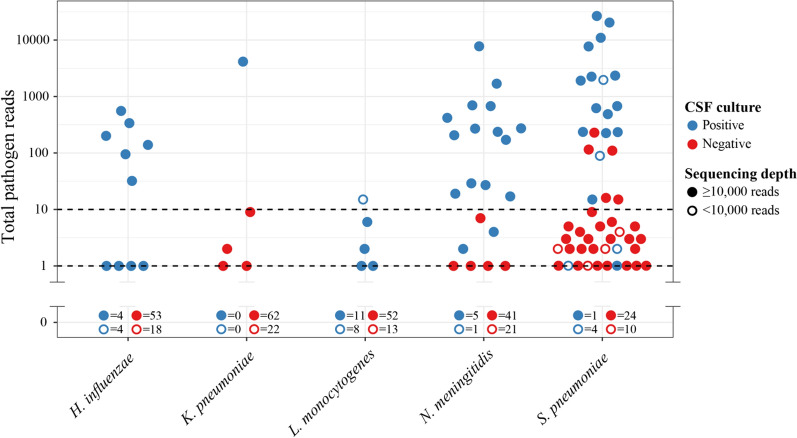
Detection of pathogen rRNA reads by VIDISCA. For each pathogen, read detection across all 89 samples is plotted (reads required ≥ 97% alignment identity to a reference sequence). Dotted lines denote the pathogen read count thresholds examined

To further understand the differences in diagnostic performance between pathogens, we produced a scatterplot of individual rRNA read counts for each, including all samples (Fig. 2). This highlighted a universally low pathogen read count for *L. monocytogenes* with a maximum of 15 reads, which likely contributes to the 21% sensitivity we observed (5 of 24 positive samples detected). Notably, 37% of *L. monocytogenes* culture positive samples were low-depth (< 10,000 reads), compared to a cohort average of 25%. Both *L. monocytogenes* and *H. influenzae* had zero false positive detections, while both *K. pneumoniae* and *N. meningitidis* had five, all with < 10 reads (Fig. 2). *S. pneumoniae* was frequently detected in samples from patients with bacterial meningitis caused by different pathogens, although 26 of 31 (84%) of these had < 10 reads and all were < 250 reads. Analysis of control CSF samples revealed similar false positive patterns (Additional file 1: Figure S1), with no *L. monocytogenes* false positives, though this time four false *H. influenzae* detections were made, all with < 10 reads. Six and two *N. meningitis* and *K. pneumoniae* false positives were found respectively (all < 10 reads). Again *S. pneumoniae* carried the highest false positive risk, with 28 false positives, 26 of which had < 10 reads, and all of which had < 19.

### Associations between clinical data and VIDISCA results

From exploratory testing, we found detection of the CSF culture diagnosed bacterium by VIDISCA was associated with no antibiotic treatment prior to presentation [49 of 78 (63%) versus 2 of 9 (22%); p = 0.03]. Detection by VIDISCA was lower in patients on immunosuppressive therapy (2 of 12 (17%) versus 48 of 79 (61%); p = 0.004), and higher in patients presenting with an altered mental status [GCS score < 14; 32 of 47 (68%) versus 19 of 42 (45%)] or a comatose state [GCS score < 8; 9 of 10 (90%) versus 42 of 79 (53%); p = 0.04]. GCS score was lower in cases accurately detected by VIDISCA, with 12 (IQR 9–15) versus 15 (IQR 12–15; p = 0.05). The level of C-reactive protein (CRP) was higher in cases detected by VIDISCA, with 206 mg/L (IQR 101–353) versus 91 mg/L (IQR 41–148; p = 0.003). The number of CSF leukocytes did not differ between groups, but CSF protein was higher in VIDISCA detected cases, with 4.1 g/L (IQR 2.9–6.0) versus 2.1 g/L (0.9–3.7; p < 0.001). No correlations between raw read count and clinical variables were found. Raw read count also did not correlate with human rRNA read count as a percentage of the total (Spearman’s rho = 0.16, p = 0.14), but the latter was weakly correlated with CSF leukocyte count (Spearman’s rho = 0.29; p = 0.006).

### Source of bacterial nucleic acids

**Fig. 3:**
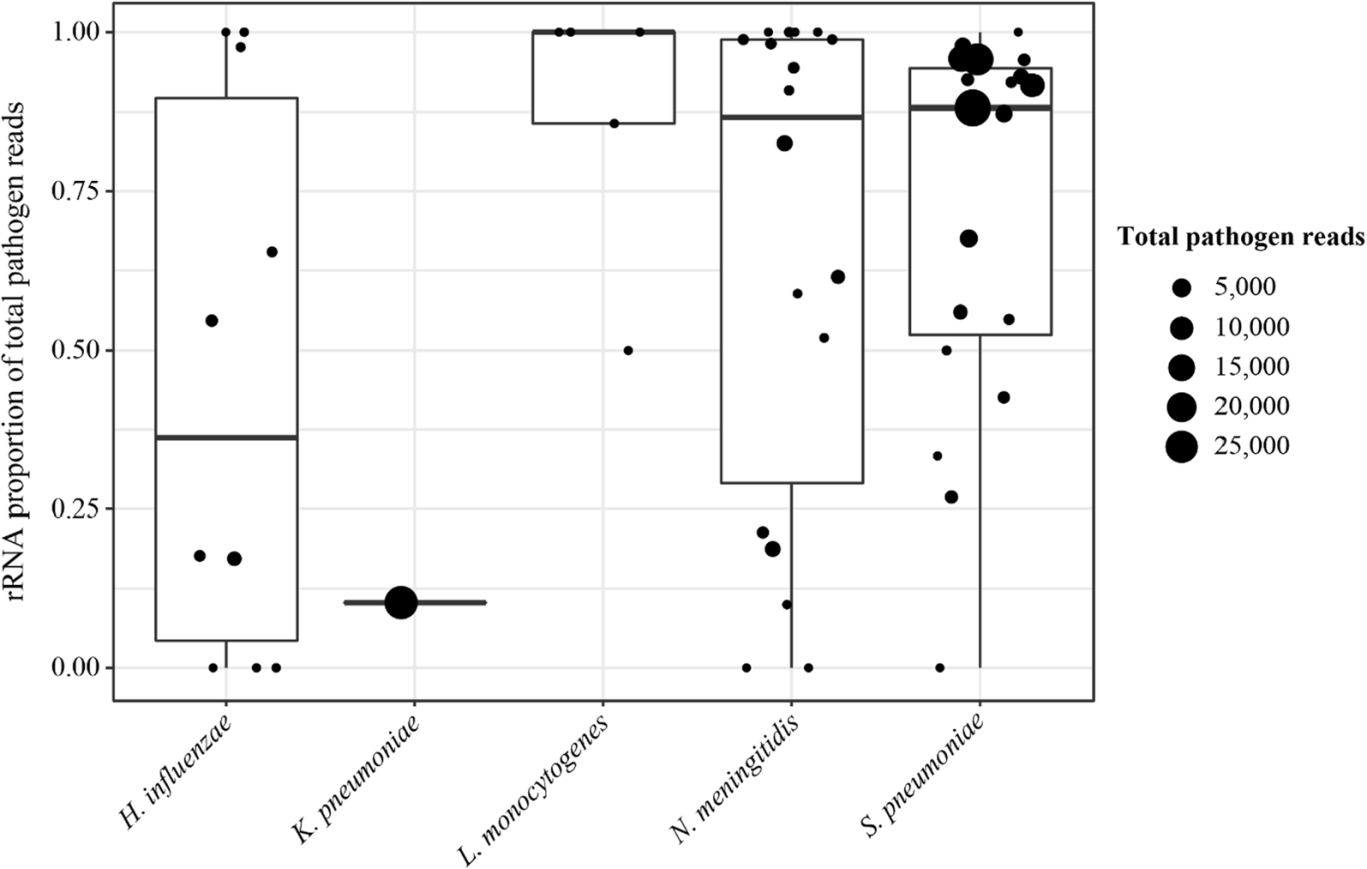
Source of bacterial reads detected by whole genome mapping. CSF culture positive samples with at least one filtered read from the respective pathogen are plotted. See also Additional file 1: Figure S2

We hypothesized bacterial reads would primarily derive from rRNA and not residual gDNA, due to our library preparation methods. Generally, rRNA did make up the major fraction of detected pathogen reads (Fig. 3), especially for *L. monocytogenes*, *N. meningitidis*, and *S. pneumoniae* samples (all with median values ≥ 87%). *S. pneumoniae* was also notable in that some samples had very high rRNA counts, which were not seen in samples containing other bacteria. Across species however, substantial read fractions from bacterial-non-rRNA were also found. In particular, *H. influenzae* had a low median value of 36% rRNA, and the single *K. pneumoniae* sample had only 10% rRNA, with a high overall bacterial-read count. To determine the likeliest source of non-rRNA reads (mRNA versus residual gDNA) we determined the proportion of non-rRNA aligning to coding sequences of respective pathogen genomes. In most cases this proportion was consistent with a predominately mRNA source, being higher than the expected value for randomly sequenced gDNA (Additional file 1: Figure S2). For *H. influenzae* however, the median proportion was precisely the expected value for gDNA, suggesting DNase treatment or centrifugation may have underperformed in CSF samples containing this species.

### Detection of viruses in CSF

Viral metagenomic analysis identified six CSF samples positive for at least one human virus (Table 2). These belonged to four families (*Flaviviridae*, *Anelloviridae*, *Picornaviridae*, and *Retroviridae*). Reads from Enterovirus D68 (EV-D68, family *Picornaviridae*) were identified in one sample, from three regions of the viral genome (Additional file 1: Figure S3A). The patient, who was taking prednisone, presented reporting gastrointestinal symptoms for several days and confusion on the day of presentation, followed by an epileptic seizure, and was diagnosed with *L. monocytogenes* meningitis. Separately, two distinct HIV-1 reads were detected in the CSF sample of a patient with meningococcal meningitis, both overlapping regions of the *Env* gene (Additional file 1: Figure S3B). Alignment to subtyped reference genomes showed the reads belonged to HIV-1 subtype B. HIV-1 infection in this patient had been discovered just prior to their admission due to meningitis, and antiretroviral therapy had not yet been started. The patient’s CD4 count was 160 cells/mm^3^, with a serum viral load of 150,000 copies/mL.

**Table 2:** Human viruses detected in patient CSF samples

Pathogen diagnosis	Virus	Read count
*L. monocytogenes*	Pegivirus A	4
*L. monocytogenes*	*Anelloviridae*	252
*L. monocytogenes*	Enterovirus D68	31
*N. meningitidis*	HIV-1 *Anelloviridae*	2 322
*H. influenzae*	*Anelloviridae*	8
*S. pneumoniae*	Pegivirus C	3

## Discussion

In patients with meningitis, no cause is found in approximately 42% of cases [30], demanding improved and broadly sensitive diagnostic methods. We demonstrate that the unmodified VIDISCA viromic method, and low-depth sequencing, can detect bacterial pathogens in CSF of patients with bacterial meningitis. Unoptimized predictive accuracy of the culture diagnosed species was low, between 38 and 51% depending on parameters, with inaccurate prediction in 3–21%. Overall sensitivity to bacterial reads was between 40 and 69% depending on threshold parameters, similar to a previous mNGS study [31], suggesting some utility as a diagnostic aid, though more suited to follow-up of undiagnosed meningitis rather than in an acute clinical setting. The performance varied substantially between pathogens, and species-specific parameter optimization improved sensitivity and specificity outcomes. Setting the threshold at a minimum of 10 pathogen reads eliminated all *K. pneumoniae* and *N. meningitidis* false positive read detections, and 26 of 31 for *S. pneumoniae*, while even one *H. influenzae* or *L. monocytogenes* read was always specific (though four negative control samples did contain up to two *H. influenzae* reads). This suggests universal cut-off criteria for diagnosis of pathogens in mNGS assays are suboptimal. Optimization by characterization of individual pathogen mNGS profiles improves the performance and utility of mNGS; however, while this is possible for common etiological agents, it is less feasible for uncommon pathogens.

A lower GCS score on presentation, no use of antibiotics, higher CSF protein levels, and higher blood CRP levels were associated with correct identification of the pathogen by VIDISCA. Several of these variables have previously been associated with increased disease severity [19, 32]. Likewise, in both clinical studies and experimental meningitis models higher bacterial loads have been shown to tightly correlate with disease severity [33]. In our study, a higher concentration of bacterial genomic material clearly influenced the likelihood of detection by mNGS. Because CSF is relatively low in genomic and protein background, we hypothesized that higher levels of background nucleic acids (proxied by CSF leukocyte count) would also increase diagnostic success by providing carrier for nucleic acid extraction. However, while CSF leukocyte count was weakly correlated with raw read count, it did not influence diagnostic success.

Apart from bacteria, we were able to identify viruses in a number of CSF samples. Some common and non-pathogenic ones were pegiviruses and members of the *Anelloviridae* [34, 35]. Viruses known to cause CNS infections were found in two samples (EV-D68 and HIV-1), from patients diagnosed with *Listeria* meningitis and meningococcal meningitis respectively. *L. monocytogenes* infection of the CNS is commonly preceded by gastrointestinal infection or colonization [36, 37], before the pathogen invades the blood and eventually crosses the blood brain barrier [38]. Although EV-D68 is primarily a respiratory virus, it may also present with gastrointestinal symptoms [39, 40]. The patient in this case was taking prednisone, and presented reporting gastrointestinal symptoms for several days. The patient appears to have been co-infected by EV-D68 and *L. monocytogenes*, although which pathogen caused the gastroenteritis is unclear. The clinical significance of HIV-1 detection in one patient is also uncertain, as the virus can often be detected in untreated HIV-1 infection without clinical signs of CNS infection [41], though it also increases the risk of bacterial meningitis by as much as eightfold compared with uninfected individuals [42].

This study has limitations. We only studied patients with bacterial meningitis confirmed by positive CSF culture, and thus the performance of VIDISCA to discriminate between different causes of infection (viral, bacterial, etc.) cannot be determined in this population. To address this, further studies in patients with suspected CNS infections should be performed. In testing the relationship between clinical variables and pathogen detection, we adopted an exploratory approach using clinical variables already known to correlate with bacterial loads, and therefore did not apply correction for multiple tests. Using data from clinically validated qPCRs would have been preferable, since this would avoid the risks of using both proxy variables and multiple statistical tests. Further, we only studied detection performance for five common pathogens in meningitis. Inclusion of additional pathogens, especially those not found in currently applied clinical rapid tests could be of particular value. Other limitations of this study may not be specific to VIDISCA, for example, the substantial number of false positive detections of *S. pneumonia* has also been observed in multiplex PCR panels [43], probably reflecting a high carriage rate in the population [44].

In conclusion, we have shown that VIDISCA is capable of detecting bacterial pathogens in CSF, mainly via rRNA. Selective depletion of human rRNA sequences enhances viral detection by VIDISCA [18], and our result implies the same effect likely applies to non-viral pathogens too. This requires rRNA sequences sufficiently distant in sequence to human rRNA, so that reverse transcription primers can anneal. VIDISCA was not developed primarily to detect bacteria, but rather as a low-depth screening method for viruses. As such, the detection rates imply that substantial improvement could be made with optimization, for example by increasing sample sequencing depth. Since VIDISCA selectively depletes gDNA, it is possible DNA-only libraries could be constructed that would enable detection of more bacterial genomic material, as has been done in other mNGS studies [8, 9]. The high overall specificity indicates that bacterial analysis of VIDISCA data is applicable in certain circumstances, for example CSF samples already being processed for viral detection and discovery, or for follow-up of idiopathic meningitis cases. The large number of samples processed (40–70) is suited to research and perhaps outbreak settings. However, current sensitivity rates and the turnaround time of between five days to two weeks make it unsuited to the acute clinical setting, where single samples will often require processing. Here, rapid and high performance multiplex assays for common pathogens are more desirable [45].

## Supplementary Information

**Additional file 1: Figure S1.** Detection of false positive reads in control CSF samples. For each pathogen, read detection across all 74 samples is plotted. Dotted lines denote the pathogen read count thresholds utilised in this study. **Figure S2.** Source of bacterial non-rRNA reads detected by whole genome mapping. The proportion of non-rRNA reads derived from bacterial coding sequence (CDS) is plotted per sample. CSF culture positive samples with at least one filtered non-rRNA read from the respective pathogen are plotted. Dotted lines represent the expected proportion of CDS reads if pure gDNA were randomly sequenced (gene density in the genome expressed as a proportion). In contrast, the expected proportion of CDS reads if pure mRNA were randomly sequenced is 1.00. Low outlier samples all had <10 total non-rRNA reads. **Figure S3.** CSF detection of neuropathogenic viruses. A) Enterovirus D68 (EV-D68) in a patient with CSF culture confirmed *Listeria* meningitis. Reads were aligned to EV-D68 strain Fermon (AY426531.1), with the position of the polyprotein open reading frame shown in black. Read matches to references are light grey, while mismatches are black. B) HIV-1 subtype B in a patient with CSF culture confirmed *N. meningitidis* infection. Reads were aligned to HIV-1 isolate WR27 (AF286365.1), with the positions of structural genes shown in black. Read matches to references are light grey, while mismatches are black.

**Additional file 2: Table S1.** Baseline characteristics of included patients. Values are n/N (%) or median (interquartile range). **Table S2.** VIDISCA performance for bacterial diagnostics.

## Data Availability

Not applicable.
