# Supplementary material for: Bacterial ribosomal RNA detection in cerebrospinal fluid using a viromics approach
Source: Fluids Barriers CNS. 2022 Dec 22;19:102. doi: 10.1186/s12987-022-00400-5 (PMC9773461; doi:10.1186/s12987-022-00400-5)
Supplement: Supplementary file 1 — Additional file 1: Figure S1. Detection of false positive reads in control CSF samples. For each pathogen, read detection across all 74 samples is plotted. Dotted lines denote the pathogen read count thresholds utilised in this study. Figure S2. Source of bacterial non-rRNA reads detected by whole genome mapping. The proportion of non-rRNA reads derived from bacterial coding sequence (CDS) is plotted per sample. CSF culture positive samples with at least one filtered non-rRNA read from the respective pathogen are plotted. Dotted lines represent the expected proportion of CDS reads if pure gDNA were randomly sequenced (gene density in the genome expressed as a proportion). In contrast, the expected proportion of CDS reads if pure mRNA were randomly sequenced is 1.00. Low outlier samples all had <10 total non-rRNA reads. Figure S3. CSF detection of neuropathogenic viruses. A) Enterovirus D68 (EV-D68) in a patient with CSF culture confirmed Listeria meningitis. Reads were aligned to EV-D68 strain Fermon (AY426531.1), with the position of the polyprotein open reading frame shown in black. Read matches to references are light grey, while mismatches are black. B) HIV-1 subtype B in a patient with CSF culture confirmed N. meningitidis infection. Reads were aligned to HIV-1 isolate WR27 (AF286365.1), with the positions of structural genes shown in black. Read matches to references are light grey, while mismatches are black. [file 12987_2022_400_MOESM1_ESM.docx]

**Supplementary information for manuscript:**

**Bacterial ribosomal RNA detection in cerebrospinal fluid using a viromics approach**

**Cormac M. Kinsella, Arthur W. D. Edridge, Ingeborg E. van Zeggeren, Martin Deijs, Diederik van de Beek, Matthijs C. Brouwer, Lia van der Hoek**

**
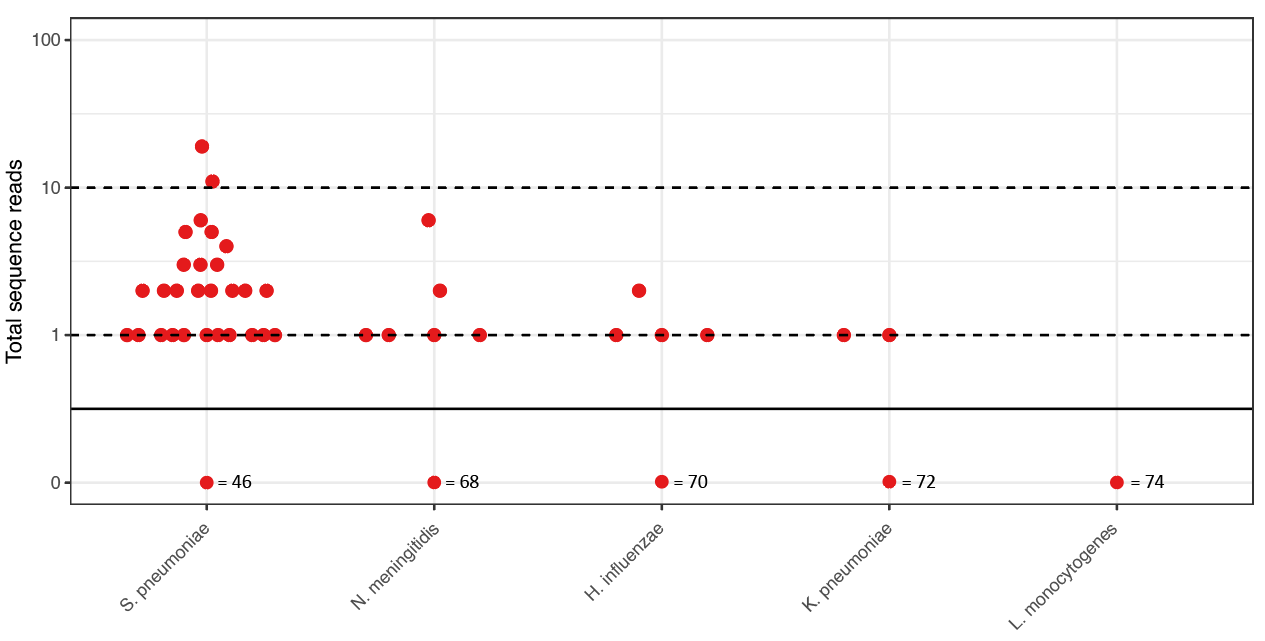
**

Figure S1. Detection of false positive reads in control CSF samples. For each pathogen, read detection across all 74 samples is plotted. Dotted lines denote the pathogen read count thresholds utilised in this study.


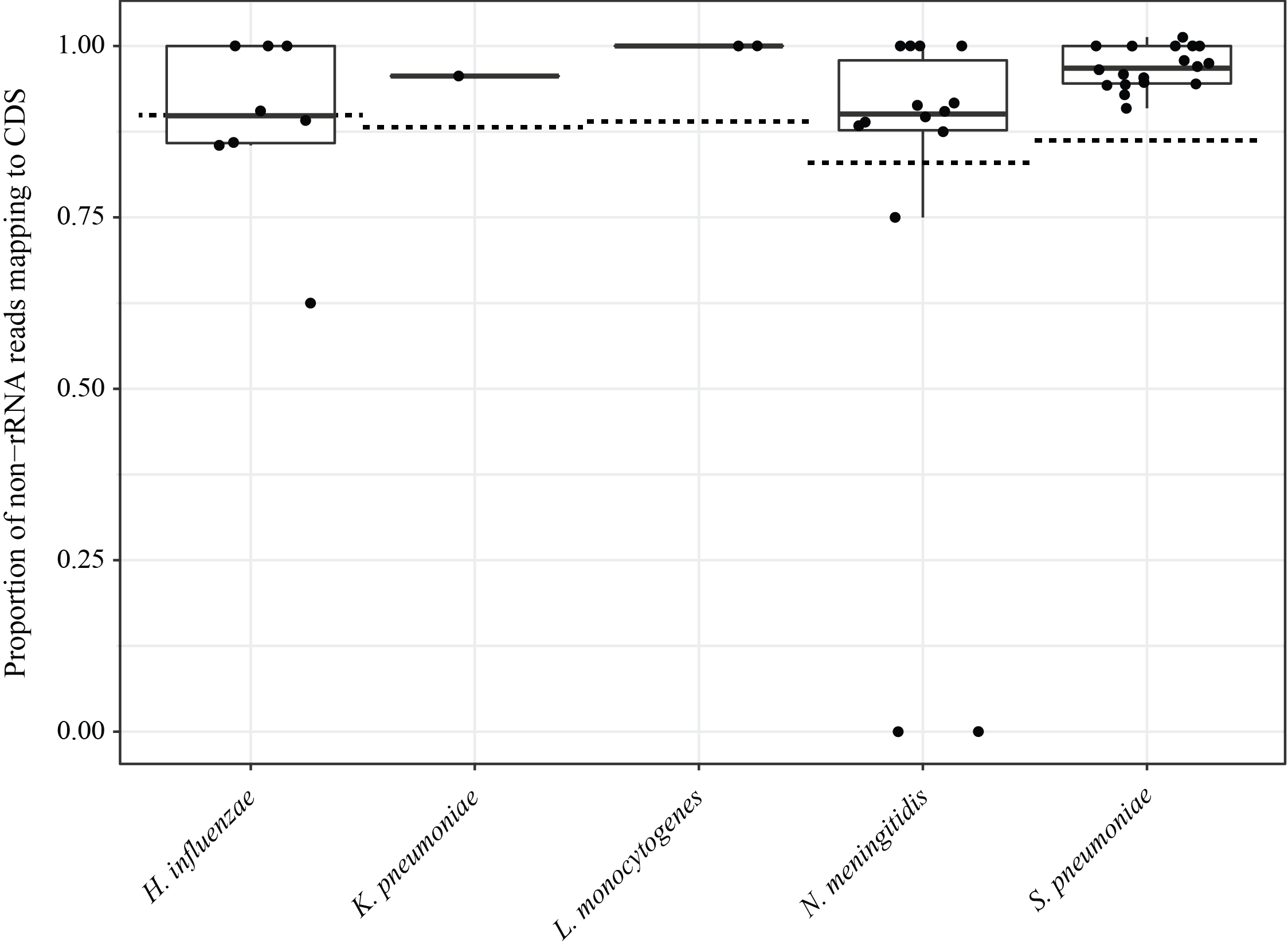


Figure S2. Source of bacterial non-rRNA reads detected by whole genome mapping. The proportion of non-rRNA reads derived from bacterial coding sequence (CDS) is plotted per sample. CSF culture positive samples with at least one filtered non-rRNA read from the respective pathogen are plotted. Dotted lines represent the expected proportion of CDS reads if pure gDNA were randomly sequenced (gene density in the genome expressed as a proportion). In contrast, the expected proportion of CDS reads if pure mRNA were randomly sequenced is 1.00. Low outlier samples all had <10 total non-rRNA reads.


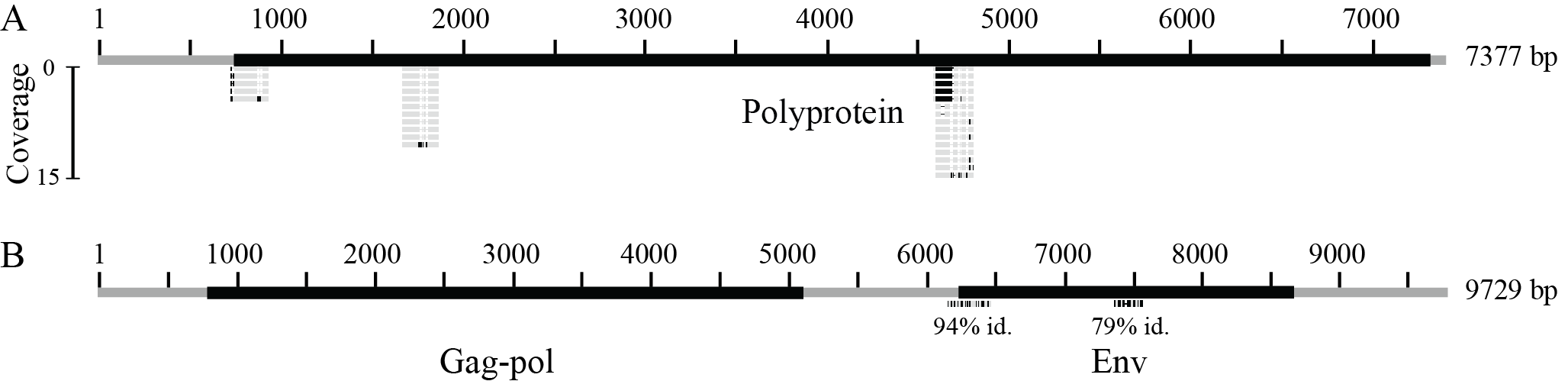


Figure S3. CSF detection of neuropathogenic viruses. A) Enterovirus D68 (EV-D68) in a patient with CSF culture confirmed *Listeria* meningitis. Reads were aligned to EV-D68 strain Fermon (AY426531.1), with the position of the polyprotein open reading frame shown in black. Read matches to references are light grey, while mismatches are black. B) HIV-1 subtype B in a patient with CSF culture confirmed *N. meningitidis* infection. Reads were aligned to HIV-1 isolate WR27 (AF286365.1), with the positions of structural genes shown in black. Read matches to references are light grey, while mismatches are black.
